# Ash and Slag Waste Processing in Self-Shielded Atmospheric DC Arc Discharge Plasma

**DOI:** 10.3390/ma15228134

**Published:** 2022-11-16

**Authors:** Zhanar Bolatova, Alexander Pak, Kirill Larionov, Dmitriy Nikitin, Pavel Povalyaev, Aleksander Ivashutenko, Gennady Mamontov, Alexey Pestryakov

**Affiliations:** 1 School of Energy & Power Engineering, Tomsk Polytechnic University, 634050 Tomsk, Russia; 2 Research Lab of Catalytic and Biomedical Technologies, Sevastopol State University, 299053 Sevastopol, Russia

**Keywords:** slag waste, coal slag, electric arc method, silicon carbide, aluminum nitride

## Abstract

In this paper, we report the experimental results obtained in slag waste processing by direct current arc discharge initiated in ambient air. The method does not employ vacuum and gas equipment, therefore increasing the energy efficiency of processing. Plasma processing of coal slag was performed at different arc exposure times: 5, 10, 15, 20, and 25 s. The obtained materials contained a significant amount of graphite, which was removed through combustion. The micropowder based on silicon carbide and aluminum nitride was obtained and then sintered by spark plasma. The bulk ceramic samples based on silicon carbide with the hardness of ~10.4 GPa were finally fabricated.

## 1. Introduction

The amount of waste generated during the combustion of coal, particularly ash and slag, is increasing due to non-stop operation of coal-fired thermal and power plants around the world [1]. Green energy technologies are actively developed, yet their energy contribution in the energy balance is still insignificant to compete with coal and coal chemistry technologies. Thus, the imperfection of coal and coal chemistry technologies and the ash and slag waste they produce cause severe environmental problems. The main components of natural coal ash include silica (up to ~61%), alumina (up to ~22%), and iron oxide (up to ~7%), which make up to ~90% of its volume. The remaining components are calcium (Ca), magnesium (Mg), sodium (Na), potassium (K), and sulfur (S) [2]. In addition to these, ash and slag often contain potentially toxic trace elements such as Hg, As, Cr, Ni, V, Se, and Cd [3,4]. Therefore, ash and slag waste should be treated as both a hazardous type of waste and a potential source of raw material for manufacturing high-demand products.

A number of currently used technologies employ ash and slag waste for the production of building materials, various structural materials, and adsorbents; less developed methods use ash and slag waste to produce geopolymers and aerogels, extract rare earth metals from ash, and manufacture catalysts [5]. It is also believed that ash and slag waste utilization can bring some metals (molybdenum, cobalt, and others) back to the production cycle in an amount comparable to the production volumes [6].

One of the approaches to ash and slag waste processing is the use of plasma to achieve the melting temperature of ash or to reduce metal and non-metal oxides in its composition. The main advantage of plasma methods is a wide range of temperatures (up to 5000–10,000 K) and heating rates up to 10^6^ K/s [7]. Materials processed using plasma methods are being used for the production of building materials [8,9] and hard ceramics [10], for the synthesis of zeolites with a high cation exchange capacity [11], and for other applications.

However, plasma methods for waste processing are not yet sufficiently developed to be widely used [12]. One of the main drawbacks is the consumption of a significant amount of electrical energy for waste processing [13], which inevitably creates waste at least during the extraction, enrichment, and processing of fuel. An important area of science and technology in the field of waste processing is related to the simplification of plasma methods, devices, and technological chains through atmospheric pressure processes [14] and the use of cheap materials as electrodes, such as natural coal or graphite [15]. An essential issue of recycling is obtaining useful ultrafine powder materials through processing [16], which implies the isolation of individual crystalline phases, particles of a given chemical composition and/or size, and separation of magnetic and non-magnetic fractions [17,18]. Thus, development of ash processing technology that employs simple equipment and consumes minimum energy to produce useful materials is of current relevance.

This paper presents the experimental results obtained during the development of an electric arc method for processing slag waste produced through the gasification of natural D-grade coal [19]. A feature of the process is its implementation in a self-shielding autonomous gaseous medium, which simplifies processing since the process does not require reactor pressurization, a vacuum pump, and the entire vacuum module. After a series of experiments, hard ceramics based on silicon carbide and aluminum nitride were fabricated.

The novelty of this study lies in the fact that for the first time, on a non-vacuum electric arc installation, slag waste was utilized to obtain ceramics based on silicon carbide.

## 2. Materials and Methods

Coal slag formed during gasification of D-grade coal was taken as the raw material [19]. The slag was crushed mechanically and then sifted through a sieve (60 µm). The resulting powder was subjected to magnetic separation to separate the slag into predominantly magnetic and non-magnetic components. The proportion of the ferromagnetic component depends on the operation mode of the coal gasifier. Published data show that silicate-type ash exhibits the highest content of magnetic iron compounds (about 10%), ash with a high aluminum content has a lower content, and calcium-rich ash contains the least amount of magnetic iron compounds [20]. The main components of the magnetic fraction of ash and slag waste are magnetite and hematite [21,22]. In this study, plasma processing of slag was performed to obtain a silicon carbide-based material; in this regard, the magnetic component must be removed from the raw material, and it requires a specific processing technology. During magnetic separation, slag was separated into powders mainly containing a ferromagnetic component and those virtually free of it. Typical XRD patterns of the initial slag and that with the magnetic fraction removed are presented in the Supplementary Materials (Figure S1). The XRD analysis showed that the initial slag and that purified from ferromagnetic fractions contain oxide crystalline phases Al_x_Si_y_O_z_, SiO_2_, and Fe_3_O_4_. In this case, the XRD pattern of the material obtained after magnetic separation showed a decreased relative intensity of the Fe_3_O_4_ diffraction maxima.

The slag micropowder purified from the magnetic fraction was mixed with carbon micropowder in a mass ratio of 2:1 using a Sample Spex Prep MixerMill8000M ball mill (SPEX, Costa Mesa, CA, USA). The resulting mixture was used as a raw material for plasma processing.

A series of experiments on processing of coal slag were performed using an experimental set-up, which was previously described in [23]. A 0.5 g (±0.001 g) sample of the raw material was placed on the crucible bottom, where it was exposed to DC arc discharge plasma at different exposure times from 3 to 25 s. The experimental technique and the set-up itself are described in more detail in the Supplementary Materials.

The samples were analyzed by X-ray diffractometry using a Shimadzu XRD 7000s X-ray diffractometer (Shimadzu, Kyoto, Japan). Qualitative analysis was performed using the ICCD PDF4+ database. Quantitative analysis was carried out by the Rietveld method.

The morphology of microsized particles of the raw material and that of the fabricated materials were analyzed using a scanning electron microscope (SEM, Vega SBU3, Tescan, Brno, Czech Republic) supplied with an energy dispersive attachment. Quantitative analysis data on the content of the main elements by the EDX method are shown in the results section in the form of a donut chart with average values for a series of measurements with a standard deviation.

Thermal analysis was carried out using a Netzsch STA 449 Jupiter analyzer (Netzsch, Selb, Germany) in an oxidizing medium. All the experiments were performed at a heating rate of 10 °C/min in a corundum crucible with a perforated lid in the temperature range of 50–1200 °C at atmospheric pressure. A sample weighing ~10 mg was placed in a mixture of air (150 mL/min) and argon (20 mL/min). According to the TG data, the determined characteristic parameters of the process were as follows: the initial temperature (T_i_) and the final temperature (T_f_) of intense oxidation; the maximum rate of the oxidation reaction (w_max_) at the corresponding temperature (T_max_); sample heating time before active oxidation (τ_e_); total active oxidation time (τ_f_).

Most of the micropowder samples were exposed to high temperatures in an oxidizing medium in an EKPS-10 muffle furnace (Lab-Term, Novosibirsk, Russia) for 1 h at 700 °C (2.8 kW electric heaters) to remove free carbon.

Consolidation of the resulting micropowder was performed by spark plasma sintering (SPS 10-4 Advanced Technologies, Newport News, VI, USA). No less than 1 g of the micropowder fabricated and purified from free carbon was loaded into graphite dies with graphite punches. The sample was sintered by heating to 1800 °C at a heating rate of 100 °C/min in vacuum at a pressure of 60 MPa and holding time of 10 min. The operating parameters of the process were taken with regard to the known literature data on the consolidation of silicon carbide-based micropowders [24].

The obtained ceramic samples were ground and polished to study the surface microstructure. Grinding and polishing were performed using a Forcipol 1 V grinding and polishing machine (diamond grinding discs: 54, 18, 6, 3 µm and polishing cloths: 6, 3, 1, 0.25 µm with diamond suspensions).

To calculate the relative density of ceramic samples, the theoretical density of ceramic samples was calculated taking into account the indicated features of the composition and the experimental density.

Hardness of the samples was measured by the Vickers method (Galileo Isoscan HV2 OD, load 1 kg).

## 3. Results and Discussions

**Figure 1:**
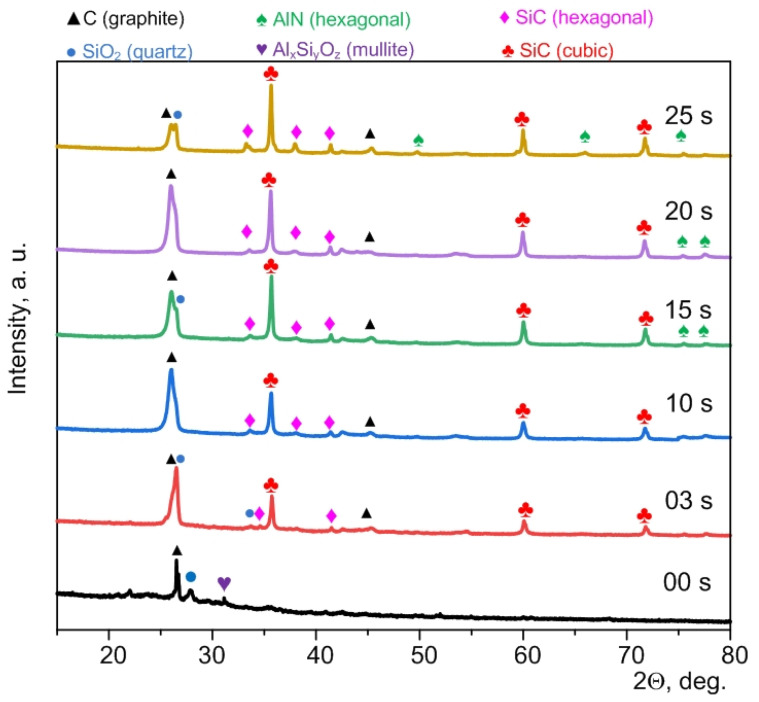
XRD patterns of the initial micropowder (00 s) and that processed by DC arc discharge plasma at different times: 3, 10, 15, 20, 25 s.

Figure 1 shows XRD patterns of the initial sample (00 s indicates 0 s plasma exposure time) and micropowders processed at different exposure times from 3 to 25 s (03 s–25 s). This time range to maintain the arc discharge corresponds to the amount of supplied energy up to 139 kJ at an average power of 4.0–4.5 kW. This is within the specific energy of electric arc processing of waste in a series of experiments equal to 139 kJ/g (by weight of the raw material).

As is known, the main components of ash and slag waste (raw material) are silicon dioxide (up to ~33.7%) and mullite (up to ~4.7%) with different carbon content (up to ~61.6%) [25]. The obtained experimental data are in line with the known literature data [26].

The diffraction patterns of the samples synthesized via the DC arc discharge method showed the following main phases: two phases of C (graphite), SiC (hexagonal), SiC (cubic), and probably AlN (hexagonal) due to the superimposition of a number of diffraction maxima. Quantitative analysis of the studied samples is indicated in the in the Supplementary Materials (Table S1). In a sample of 25 s, a decrease in the graphite phase can be observed compared to the rest of the samples; this is explained by the fact that with a duration of synthesis of 25 s, a lot of powder based on silicon carbide and graphite sinter are formed in the crucible, which was removed mechanically. The two carbon phases are formed due to electrical erosion of the anode with mass transfer from the anode to the cathode [27] and the presence of residual carbon in the raw material. The absence of mullite peaks (Al_x_Si_y_O_z_) and the reduced amount of quartz SiO_2_ indicate their consumption during the formation of SiC [26,27] and AlN [28] phases via carbothermal reduction. The data reported in [29,30,31] confirm the formation of silicon carbide phases by electric arc processing in air. In addition, the XRD pattern shows low-intensity traces of crystalline phases of hexagonal aluminum nitride. Superimposition of the main diffraction maxima of aluminum nitride and the maxima of silicon carbide complicates accurate phase identification by X-ray diffractometry. Yet, in addition to silicon carbide, aluminum nitride can be formed during electric arc processing of ash and slag waste [32].

Silicon carbide and aluminum nitride can be formed according to the known reaction equations.

SiO_2_ (s) + 3C (s) = SiC (s) + 2CO (g) (1)

Al_2_O_3_ + 3C + N_2_→2AlN + 3CO (2)

The main carbide phase is cubic silicon carbide with lattice parameters a = 4.3562 Å ± 0.0002 (estimated in a series of experiments). A hexagonal phase of silicon carbide with lattice parameters a = 3.0802 Å ± 0.0201 Å and c = 15.1434 Å ± 0.0044 Å can be identified as well. Within the limits of possible errors, these values of the lattice parameters are in good agreement with those of the reference phases no. 00-900-8856 and no. 00-154-1661 from the ICCD PDF4+ database and with the data published in [33]. The presence of two silicon carbide phases in the products of electric arc synthesis of silicon carbide is expected due to the results reported in [32].

According to the XRD results, the reduction of oxide phases in slag waste occurred during arc discharge burning. In this case, all the synthesized products are contaminated with the graphite phase, which is due to the use of graphite electrodes and electroerosion processes [27,34]. The study then evaluated the feasibility of synthesized products purification from free carbon via powder combustion in an atmospheric furnace. Differential thermal analysis was carried out to estimate the characteristic temperature intervals.

**Figure 2:**
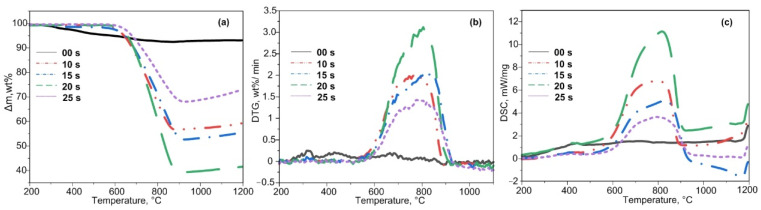
TG (**a**), DTG (**b**), and DSC (**c**) curves for oxidation of the initial ash and that processed in the air–argon mixture at a heating rate of 10 °C/min in the temperature range of 25–1000 °C.

Experimental TG, DTG, and DSC curves for oxidation of the test samples are presented in Figure 2. Experimental data of the TG analysis show that an exothermic process begins at about 600 °C, ends at 900 °C, and is accompanied by weight loss. The greatest weight loss can be observed in samples processed for 20–25 s, where it amounts to ~60%. The weight loss for both 10 s and 15 s samples is ~40–45%. The smallest weight loss is observed for the sample at 25 s, since the graphite sinter was removed mechanically; this is confirmed by X-ray diffractometry in Figure 1. The initial sample (00 s) does not exhibit a pronounced exothermic weight loss, which can be explained by a different proportion of graphite in the materials (the initial slag does not contain graphite, the proportion of graphite is higher in the samples with a longer exposure time).

The temperature of the intense oxidation onset is different for each sample. For plasma-processed samples (10–20 s), the T_i_ values were significantly higher and varied in the range of 268–413 °C (Table 1).

Similar to the T_i_ parameter, the T_f_ (Table 1) varied for all samples, except for the 20 s sample, which exhibited the maximum value of 898 °C.

The DTG data (Figure 2b) showed a monomodal peak in the temperature range of 500–950 °C for the initial sample oxidation. The 15 s sample did not show monomodal peaks. The oxidation of the 20 s sample was characterized by a maximum reaction rate w_max_, which amounted to 2.02 wt %/min (Table 1). For other samples, the average value of this parameter was 1.81 wt %/min.

The DSC analysis shows (Figure 2c) that the synthesized samples undergo an exothermic process at 600–850 °C. For example, the greatest heat release was observed in the 20 s sample at 825 °C, which correlates with the operation mode of the arc reactor (more eroded graphite enters the synthesized product at a longer exposure time). After plasma processing, the calculated value of the integrand area of the DSC curves was observed to increase at increased carbon plasma exposure time (Table 1) [33].

The DTA data revealed characteristic temperatures suitable for purification of the synthesized products from excess uncombined carbon through its combustion. Silicon carbide-based micropowders were purified from excess carbon through the material combustion in an atmospheric furnace. With regard to the thermal analysis data and literature data, the heating mode was chosen to be 1 h exposure at 700 °C.

**Figure 3:**
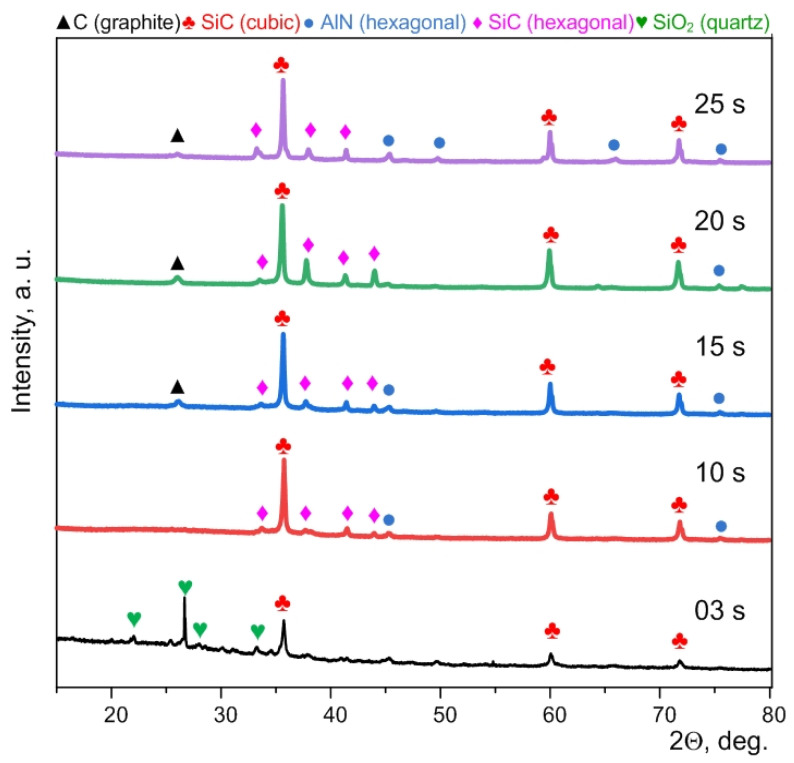
XRD patterns of the synthesized products purified from excess carbon and obtained at different times.

XRD patterns of the synthesized micropowders purified from carbon confirm the formation of cubic and hexagonal phases of silicon carbide (Figure 3). Quantitative data for these samples are indicated in the Supplementary Materials (Table S2). In addition, graphite and quartz peaks can be identified. The presence of graphite is probably due to unburned residual graphite in the volume of particle agglomerates.

The presence of separate weak traces of quartz can be due to incomplete electric arc processing of the initial powders; in addition, surface oxidation of silicon carbide can occur during purification from graphite [27].

**Figure 4:**
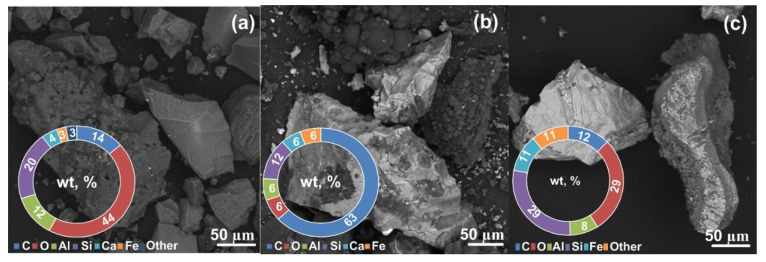
SEM results for the initial slag (**a**), the synthesized product (**b**), and the carbon-free product (**c**).

Figure 4a presents the results of scanning electron microscopy and energy dispersive analysis of the initial slag sample. The scanning electron microscopy of the initial sample revealed the presence of irregularly shaped crystalline particles. The spectra of the initial sample showed a significant amount of oxygen, silicon, carbon, aluminum, and a small amount of calcium, iron, sodium, magnesium, and some other elements with a content close to zero, which is characteristic of ash and slag waste samples [35], since ash and slag waste are inhomogeneous and vary in composition depending on the coal origin. Nevertheless, the literature data report the characteristic features of the qualitative composition of slag. Oxygen, aluminum, and silicon are the main components of ash. Slag also contains carbon, sodium, phosphorus, sulfur, potassium, calcium, titanium, iron, and some other elements in fractions of a percent [35,36]. Analysis of the data obtained in a series of EDX measurements of the chemical composition of the initial slag is shown in Figure 4a. The figure presents quantitative data on the content of the main elements, such as O, Al, Si, Ca, and Fe, in the form of a donut chart with averaged values for a series of measurements with a standard deviation. Na, Mg, P, S, and K were also identified in the material. The content of Ti, Mn, and Cu was negligible (not more than 1 wt %).

For the 25 s sample (Figure 4b), the SEM data revealed the presence of solid particles in the form of irregularly shaped crystalline particles.

Analysis of the energy dispersive spectra of plasma-processed samples revealed a significant amount of carbon, silicon, and oxygen and a small amount of aluminum, calcium, iron, and some other elements with a content close to zero. For the samples processed at the highest energy and purified from free carbon (Figure 4c), the SEM data revealed irregularly shaped ash particle agglomerates with sizes of up to several hundred micrometers. The energy dispersive spectra of these samples showed an increased amount of silicon and a decreased amount of carbon, which correlate with the XRD data; in this case, the fraction of oxygen averaged over a series of measurements decreases, and in a series, it can vary locally from 0% to 50%.

**Figure 5:**
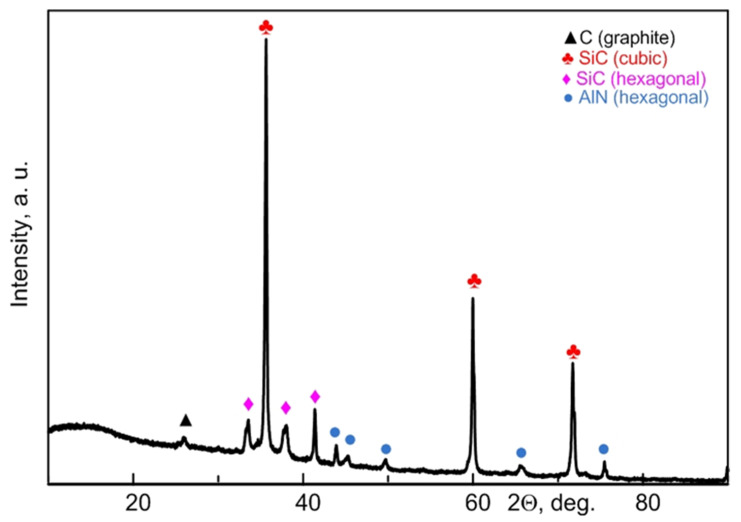
XRD pattern of silicon carbide-based micropowder obtained in the recommended mode (220 A, 25 s) and purified from excess carbon.

After a series of experiments performed based on the above data, the electric arc processing mode (current strength 220 A, arc duration 25 s) was chosen for the production of the raw material for subsequent studies, and it was purified from excess carbon. Figure 5 shows the XRD pattern of the sample accumulated for subsequent sintering. According to the XRD data, the sample contains hexagonal and cubic phases of silicon carbide, a hexagonal phase of aluminum nitride, and traces of carbon.

The resulting material was sintered by spark plasma without sintering additives. For comparison, commercial silicon carbide micropowder was sintered under similar conditions without and with sintering additives (aluminum, boron, carbon). Table 2 summarizes the main sintering parameters and results. The experimental technique and the setup itself are described in more detail in the Supplementary Materials (Figure S2).

**Figure 6:**
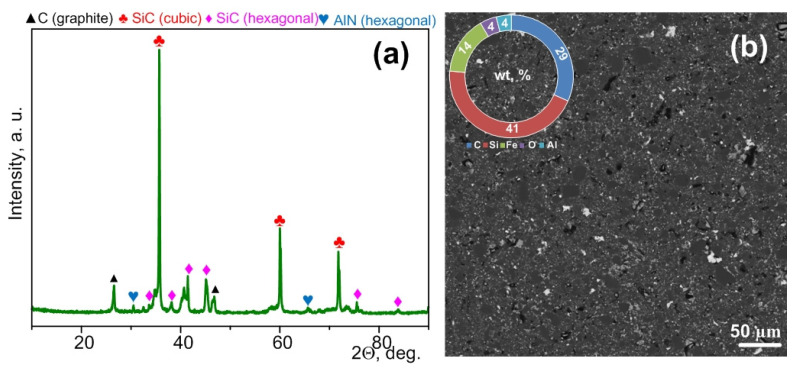
XRD pattern of the bulk ceramic sample sintered from silicon carbide-based micropowder by spark plasma sintering (**a**), SEM image of a section of the sintered sample surface and data of the EDS analysis (**b**).

According to the XRD analysis (Figure 6a), the resulting material contains up to ~49.6 vol % of cubic silicon carbide, up to ~31.6 vol % of hexagonal silicon carbide, up to ~18.8 vol % of graphite, and peaks of aluminum nitride (Figure 6a). It should be noted that traces of silicon and iron oxides can be seen in the XRD pattern and SEM images of the obtained materials based on silicon carbide (Figure 6b).

It should be noted that the relative density of the resulting material was 94.7%, which is significantly higher than that of the sample sintered from commercial silicon carbide without sintering additives (with similar parameters), and it was close to the density of the sample sintered from commercial silicon carbide with sintering additives (Table 2). At the same time, the sample obtained from waste contains impurities of various metals, which overestimate the calculated density. Averaged hardness measurements showed that the hardness of the sample obtained from waste is lower than that of samples sintered from commercial silicon carbide both with and without sintering additives. The hardness of the material obtained from waste is 25% lower than that of commercial silicon carbide micropowder sintered under similar conditions. Thus, the quality of the resulting ceramic is apparently lower than that of the ceramic obtained from commercial raw materials; however, the obtained material is of great value due to its production via waste processing using a simple technique but not due to its high quality.

## 4. Conclusions

This paper presents the results of slag waste processing by DC arc discharge plasma. During processing, silicon carbide-based micropowder was obtained. The resulting micropowder contained free carbon, which was subsequently removed via the micropowder combustion in an atmospheric furnace at 700 °C for 1 h. In a series of experiments, a plasma processing mode (200 A, 25 s) was chosen to produce raw materials for subsequent sintering. As a result of spark plasma sintering of the synthesized material, bulk ceramic samples based on silicon carbide were obtained with the hardness of 10.3 ± 0.4 GPa, which is lower compared to that of commercial silicon carbide. The experimental data showed that slag waste utilization via the DC arc discharge plasma method can be employed to obtain silicon carbide-based micropowders.

## Figures and Tables

**Table 1:** Parameters of the test sample oxidation.

Parameter	00 s	10 s	15 s	20 s	25 s
Initial temperature of intense oxidation, T_i_, °C	263	268	320	395	322
Final temperature of intense oxidation, T_f_, °C	848	883	923	898	926
Maximum reaction rate, w_max_, wt %/min	0.24	2.00	2.02	3.05	1.42
Temperature of the maximum reaction rate, T_max_, °C	315	775	825	810	793
Time of attaining the maximum reaction rate, T_max_, min	26.5	75.5	77.5	76.0	74.5
Time of sample heating before active oxidation, T_e_, min	21.0	21.5	26.5	34.0	27.0
Total time of active oxidation, τ_f_, min	58.5	61.5	60.3	50.3	60.4
Area of the DSC curve	360.8	9085	7208	11232	5470

**Table 2:** Sintering parameters and results for commercial silicon carbide and for that obtained through slag plasma utilization.

Sample	Sintering Parameters				ρ, g/cm^3^	ρ, %ρ_th_	H, GPa
	T, °C	P, MPa	ΔT/Δt, K/min	Δt, min			
SiC (TSPROF F230, Russia) (from commercial raw materials, the current work)	1800	60	100	10	2.25	70.0	2.2 ± 0.6
SiC (from slag, the current work)					3.04	95.2	10.3 ± 0.4
SiC + Al (4%) + B (2%) + C (2%) [25]					3.03	95.3	23.3 ± 0.3
SiC [37]	1800	40	373	5	-	87.2	10.2
SiC [38]	1860	50	423	5	-	98.5	28.5
SiC [39]	1850	75	373	10	2.58	80.0	-
SiC–B4C +Al (8%) [40]	1800	40	-	-	-	-	26.20

## Data Availability

Data are available on request from the authors.

## Supplementary Materials

The following supporting information can be downloaded at: https://www.mdpi.com/article/10.3390/ma15228134/s1, Figure S1. Typical X-ray diffraction patterns: initial coal slag and slag after magnetic separation. Figure S2. The sintering process for commercial silicon carbide. Table S1: Quantitative XRD data of the initial micropowder (00 s) and that processed by DC arc discharge plasma at different times: 3, 10, 15, 20, 25 s. Table S2: Quantitative XRD data of the synthesized products purified from excess carbon and obtained at different times.
