# Supplementary material for: Ash and Slag Waste Processing in Self-Shielded Atmospheric DC Arc Discharge Plasma
Source: Materials (Basel). 2022 Nov 16;15(22):8134. doi: 10.3390/ma15228134 (PMC9698856; doi:10.3390/ma15228134)
Supplement: Supplementary file 1 [file materials-15-08134-s001.zip › materials-1979715-supplementary.pdf]

Supplementary Materials

# Ash and Slag Waste Processing in Self-Shielded Atmospheric DC Arc Discharge Plasma

**Zhanar Bolatova [1,*], Alexander Pak [1], Kirill Larionov [1], Dmitriy Nikitin [1], Pavel Povalyaev [1], Aleksander Ivashutenko [1], Gennady Mamontov [1] and Alexey Pestryakov [1,2,*]**

[1] School of Energy & Power Engineering, Tomsk Polytechnic University, 634050 Tomsk, Russia
[2] Research Lab of Catalytic and Biomedical Technologies, Sevastopol State University, 299053 Sevastopol, Russia
* Correspondence: zsb3@tpu.ru (Z.B.); pestryakov@tpu.ru (A.P.); Tel.: +7-(3822)-606-117 (A.P.)

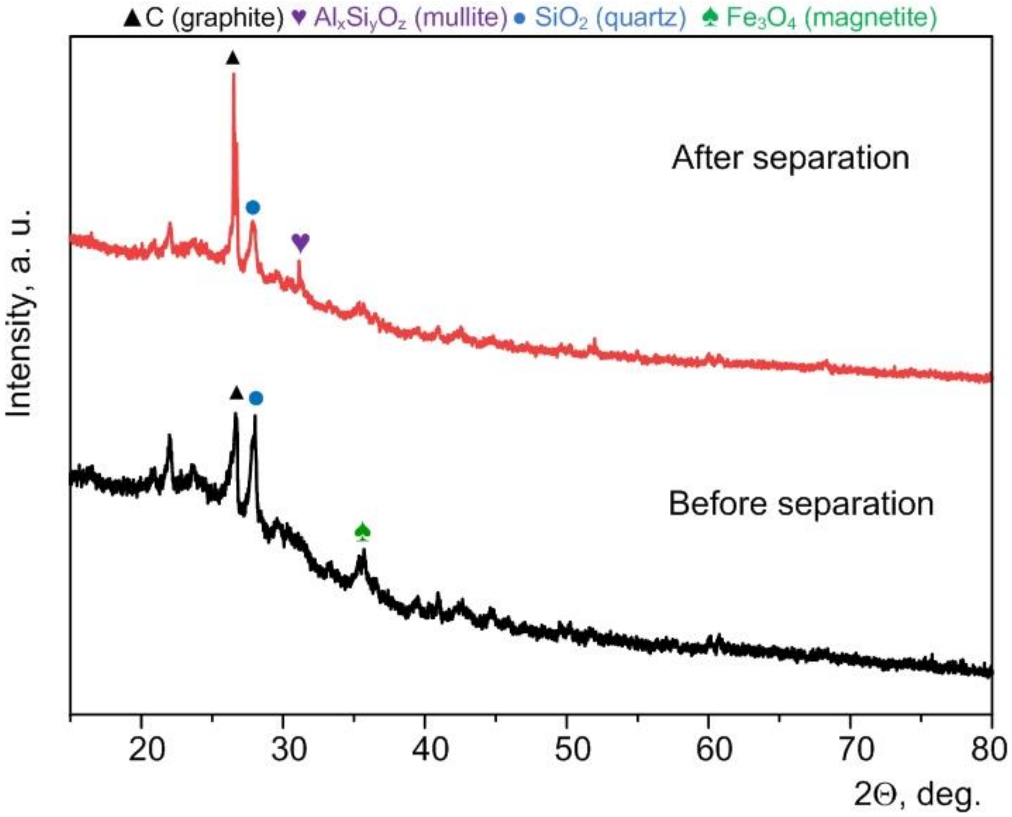


**Figure S1.** Typical X-ray diffraction patterns: initial coal slag and slag after magnetic separation

## S1. Experimental Setup

A series of experiments on processing of coal slag was performed using an experimental setup, which provides the effect of self-shielding of the reaction volume from atmospheric oxygen during plasma processing of raw materials [23]. The technique employs the temperature of carbothermal reduction of oxide phases attained due to the thermal field of the arc discharge. In this case, when arc discharge is initiated in air, the working volume of the reactor is shielded from atmospheric oxygen due to the formation of CO and CO2 gases in the reaction zone. As a result, when arc discharge is initiated in air and the conditions for self-shielding of the reaction volume are attained, reduction processes can be realized with the formation of non-oxide compounds [23]. The electric arc reactor employs a power source with a step-up frequency converter, a step-down power transformer, and a controlled rectifier. The operating current of the power source was 200 A at open circuit voltage of 63 V. Graphite electrodes were connected to the power source. The anode was a graphite rod with a diameter of 8 mm; a graphite crucible with a diameter

of 30 mm, a height of 30 mm, and an internal cavity diameter of 20 mm was used as a cathode. The cathode was fixed immobile by the current-carrying clamps of the power source; the anode was moved by a vertical linear electric drive with a stepper motor was mounted above the cathode. The discharge gap was set equal to 0.5 mm. The stepper motor in the electric drive was controlled by the controller. The current in the discharge circuit and the arc discharge voltage were measured using an oscilloscope connected to the electrodes (through a step-down ohmic voltage divider and a current sensor). The TEST 1 probe gas analyzer (Boner, Russia) installed above the cathode analyzed the autonomous gaseous medium. A 0.5 g (± 0.001 g) sample of the raw material was placed on the crucible bottom, where it was exposed to DC arc discharge plasma at different exposure times: from 3 to 25 s.

**Table S1**: Quantitative XRD data of the initial micropowder (00 s) and that processed by DC arc discharge plasma at different times: 3, 10, 15, 20, 25 s.

| | $SiC_{hex}$ | $SiC_{cub}$ | $SiO_2$ | C | AlN | $Al_xSi_yO_z$ |
|---|---|---|---|---|---|---|
| **00 s** | - | - | 33.7 | 61.6 | - | 4.7 |
| **03 s** | 7.4 | 9 | 3 | 80.5 | | |
| **10 s** | 9.4 | 9.4 | - | 81.2 | traces | - |
| **15 s** | 7.8 | 18.8 | - | 71.6 | 1.7 | - |
| **20 s** | 10.8 | 11.5 | - | 77.7 | traces | - |
| **25 s** | 11 | 19 | - | 62.6 | 7.4 | - |

**Table S2:** Quantitative XRD data of the synthesized products purified from excess carbon and obtained at different times

| | $SiC_{hex}$ | $SiC_{cub}$ | $SiO_2$ | C | AlN |
|---|---|---|---|---|---|
| **03 s** | traces | 65 | 23.5 | traces | 11.5 |
| **10 s** | 30.1 | 44.9 | - | 2.3 | 22.7 |
| **15 s** | 34.9 | 44.5 | - | 17 | 3.6 |
| **20 s** | 21.6 | 72.5 | - | 2.4 | 3.5 |
| **25 s** | 25.1 | 54.7 | - | 62.6 | 10.4 |

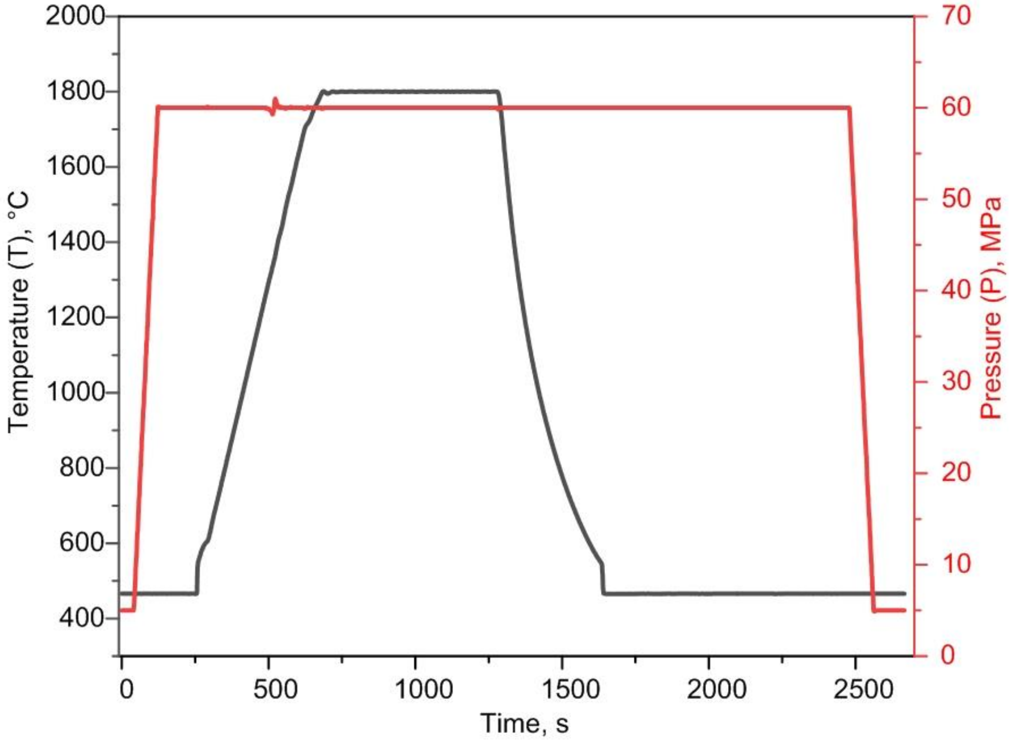


**Figure S2.** The sintering process for commercial silicon carbide

**References**


23. Pak, A.Y.; Shanenkov, I.I.; Mamontov, G.Y.; Kokorina, A.I. Vacuumless Synthesis of Tungsten Carbide in a Self-Shielding Atmospheric Plasma of DC Arc Discharge. *Int. J. Refract. Met. Hard Mater.* **2020**, *93*, 105343. https://doi.org/10.1016/j.ijrmhm.2020.105343.
